# Thyroid Stimulating Hormone Is Increased in Hypertensive Patients with Obstructive Sleep Apnea

**DOI:** 10.1155/2016/4802720

**Published:** 2016-11-02

**Authors:** Nanfang Li, Mulalibieke Heizhati, Chao Sun, Suofeiya Abulikemu, Liang Shao, Xiaoguang Yao, Yingchun Wang, Jing Hong, Ling Zhou, Lei Wang, Yu Zhang, Weiwei Zhang

**Affiliations:** The Center for Hypertension of the People's Hospital of Xinjiang Uygur Autonomous Region, The Center for Diagnosis, Treatment and Research of Hypertension in Xinjiang, No. 91, TianChi Road, Urumqi, Xinjiang 830001, China

## Abstract

*Purpose*. To evaluate alteration in serum TSH in hypertensives with OSA and its relation with cardiometabolic risk factors.* Methods*. 517 hypertensives were cross-sectionally studied. OSA was determined by polysomnography and thyroid function by standard methods.* Results*. OSA was diagnosed in 373 hypertensives (72.15%). Prevalence of subclinical hypothyroidism was significantly higher in OSA hypertensives than in non-OSA ones (15.0% versus 6.9%, *P* = 0.014). Serum LnTSH in hypertensives with severe OSA was significantly higher (0.99 ± 0.81 versus 0.74 ± 0.77 *μ*IU/mL, *P* < 0.05) than in those without OSA. AHI, LSaO_2_, ODI_3_, and ODI_4_ were independently associated with serum TSH for those aged 30–65 years. Dividing subjects into four groups as TSH < 1.0 *μ*IU/mL, 1.0 ≤ THS ≤ 1.9 *μ*IU/mL, 1.91 ≤ TSH < 4.5 *μ*IU/mL, and TSH ≥ 4.5 *μ*IU/mL, only 26.3% of OSA subjects exhibited TSH between 1.0 and 1.9 *μ*IU/mL, significantly less than non-OSA subjects (26.3% versus 38.2%, *P* = 0.01). DBP and serum LDL-c elevated with TSH increasing and were only significantly higher in TSH ≥ 4.5 *μ*IU/mL group than in 1.0 ≤ TSH ≤ 1.9 *μ*IU/mL group (96.32 ± 14.19 versus 92.31 ± 12.86 mmHg; *P* = 0.040; 0.99 ± 0.60 versus 0.87 ± 0.34 mmol/L, *P* = 0.023).* Conclusion*. OSA might be a risk factor for increased TSH even within reference range in hypertensive population.

## 1. Introduction

Obstructive sleep apnea (OSA) is characterized by an intermittent repeatable cessation of airflow to the lung due to closure of the airway at pharyngeal level and afflicting about 24–42% adults and has been implicated as an independent risk factor for hypertension, cardiocerebrovascular diseases, and metabolic complications [1, 2].

Accumulating evidence suggests a bidirectional relationship between OSA and thyroid dysfunctions, clinical and subclinical hypothyroidism in particular, which is well established to correlate with atherosclerosis, hypertension, cardiovascular diseases, and metabolic disorders [3–5]. It has been clearly evidenced that hypothyroidism leads to OSA via giving raise to mucoprotein deposition in the upper airway, decreased neural output to airway musculature, obesity, and abnormal ventilatory control since the first description in 1964 [6, 7].

On the other hand, OSA may also have consequences on hormonal axis and might be linked to or even cause hypothyroidism [8–10], whereas studies have been completed with conflicting conclusions on the strength of the association between OSA and hypothyroidism [11–14]. A recent study showed no difference in prevalence of subclinical and clinical hypothyroidism in OSA patients, compared to general subjects [11], and consistent results were presented by Resta and colleagues [12, 15]. However, Young and colleagues reported that Hashimoto's thyroiditis, the most frequent reason of hypothyroidism [1], accounted for almost half of OSA patients (46.8%) and its prevalence was parallel to OSA severity [13]. Furthermore, Resta and colleagues showed that prevalence of previously undiagnosed subclinical hypothyroidism is 11.5% in 78 overweight and obese OSA subjects with no previous hypothyroidism [14], supported by a recent study exhibiting higher prevalence of newly diagnosed subclinical hypothyroidism (11.1%) in OSA adults, but no difference was found in prevalence of clinical hypothyroidism between OSA and control subjects [16]. Of note, most researchers hold consistent viewpoint that individuals with OSA should be referred to thyroid function evaluation [11–16].

Clinical overlap between OSA and hypothyroidism creates a significant risk of undiagnosed hypothyroidism in OSA. As a matter of fact, however, the incidence of thyroid deficiency status in OSA patients is not clearly apprehended, missing the diagnosis of hypothyroidism in OSA may lead to inappropriate or unnecessary nocturnal continuous positive airway pressure treatment and may also lead to increased severe complications of OSA via aggregating cardiometabolic risk profiles, and missing the diagnosis of OSA in hypothyroid patients may lead to an increased morbidity and mortality due to increasing cardiometabolic risk for OSA [3–5, 17]. Therefore, we conducted a cross-sectional study to evaluate the association of OSA and alteration in serum TSH and its correlation with cardiometabolic risk profiles in hypertensive population hospitalized for high blood pressure.

## 2. Subjects and Methods

### 2.1. Study Subjects

Patients hospitalized in the center for diagnosis, treatment, and research of hypertension at People's Hospital of Xinjiang Uygur Autonomous Region from Jan to Dec 2010 were selected, based on the following exclusion and inclusion criteria. Exclusion criteria: subjects with a history of long-term alcohol-intake, asthma, bronchiectasis, severe maxillary facial deformities, preexisting thyroid diseases, chronic obstructive pulmonary diseases, acute phase of infection, history of upper-airway surgery, history of pancreatic surgery, pheochromocytoma, primary hyperaldosteronism, established T2DM or taking hypoglycaemic drugs, cerebrocardiovascular diseases within 6 months, severe hepatic and/or renal diseases, and cancer were excluded from this study. Inclusion criteria: patients who had nocturnal snoring, apneas, and diurnal sleepiness during reading, watching TV, or meetings lasting at least an hour and after the meal (without drinking) via medical history and physical examination and patients with unexplained lip and/or tongue dryness, unexplained cyanosis of lip and/or nail bed, and elevation of hemoglobin levels and/or hematocrit were included. Eventually, 517 hypertensive inpatients constituted study subjects. Study protocol was approved by Ethics' Committees of Health Ministry in Xinjiang Uygur Autonomous Region and abovementioned hospital; informed consent was signed by all subjects.

### 2.2. Demographic and Clinical Measurements

Blood pressure measurements were performed manually 3 times using calibrated mercury sphygmomanometers in the morning by trained nurses of hypertension center; and the mean of the last 2 measurements was recorded. New-onset hypertension was defined as systolic blood pressure (SBP) ≥ 140 mmHg and/or diastolic blood pressure (DBP) ≥ 90 mmHg, measuring blood pressure three times in different days. Known hypertension was defined as hypertension or self-reported intake of antihypertensive drugs [18]. Height and weight of all patients were measured using standardized equipment during the hospitalization.

### 2.3. OSA Evaluation

All participants underwent overnight attended polysomnography (PSG). All subjects were required not to take coffee, alcohol, and sedative hypnotic drugs prior to sleep study. PSG evaluation included airflow monitoring with thermocouple and/or nasal pressure, respiratory effort using piezo belts at the chest and abdominal positions, oxygen saturation using pulse oximetry, surface electrodes attached using standard techniques to obtain an electrooculogram, and electromyogram of the chin. Sleep stages were defined according to Rechtshaffen and Kales' criteria by a professional polysomnographic technologist. A reduction in the amplitude of airflow of at least 30% for ≥10 s followed by either a decrease in oxygen saturation of 4% or a reduction in the amplitude of airflow of at least 50% for ≥10 s followed by either a decrease in oxygen saturation of 3% or signs of physiologic arousal composes the definition of hypopnea. Apnea hypopnea index (AHI), lowest saturation of oxyhemoglobin (LSaO_2_), oxygen desaturation index of 3% (ODI_3_), and oxygen desaturation index of 4% (ODI_4_) during sleep were calculated in each patient. OSA severity was defined by AHI as follows: non-OSA (AHI < 5), mild OSA (5 ≤ AHI < 15), moderate OSA (15 ≤ AHI < 30), and severe OSA (AHI ≥ 30) [19].

### 2.4. Thyroid Function Evaluation

In accordance with routine clinical procedure, 5 mL fasting venous blood samples were collected at 07:00-08:00 am in the morning after overnight fasting and centrifuged (10 min 3000/min) 30 min after put in room temperature and biochemical evaluations were performed at the clinical laboratory of the People's Hospital of Xinjiang Uygur Autonomous Region. Biochemical evaluation including serum thyroid stimulating hormone (TSH), serum three iodine thyroid original amino acid (TT_3_), serum thyroxine (TT_4_), free triiodothyronine (FT_3_), free thyroxine (FT_4_), antithyroglobulin (ATG), and anti-thyroid peroxidase (TPO) was measured using electrochemiluminescent immunoassay (Roche cobas e601 electrochemical luminescence analyzer, Roche Germany). Reference ranges for thyroid tests were 0.27–4.2 *μ*IU/mL for TSH, the intra-assay coefficients of variation %, 0.8–2.0 ng/mL for TT_3_, 5.1–14.1 ng/mL for TT_4_, 2.0–4.4 pg/mL for FT_3_, 0.93–1.7 ng/dL for FT_4_, <116 IU/mL for ATG, and <35 IU/mL for TPO.

Thyroid status was defined as follows [20]:

*Euthyroid*. Serum TSH and FT_4_ were within the normal range, 0.40–4.5 *μ*IU/mL and 0.93–1.70 ng/dL, respectively.
*Overt Hypothyroid*. Serum TSH was ≥ 4.5 *μ*IU/mL and FT_4_ < 0.93 ng/dL.
*Subclinical Hypothyroidism*. Serum TSH was ≥ 4.5 *μ*IU/mL and FT_4_ within normal range: 0.93–1.70 ng/dL.
*Overt Hyperthyroid*. TSH was < 0.40 *μ*IU/mL; FT_4_ > 1.7 ng/dL; FT_3_ > 4.4 pg/mL, or both.
*Subclinical Hyperthyroid*. TSH was < 0.40 *μ*IU/mL; FT_3_ and FT_4_ were within the normal ranges: 2.0–4.4 pg/mL and 0.93–1.7 ng/dL.

### 2.5. Statistical Analysis

Before statistical analysis, normal distribution and homogeneity of the variances were evaluated using Levene's test. Data are expressed as percentage or means ± SD if they are normally distributed and data are first log transformed if not normally distributed. Data not log transformed are expressed as median (quartile range). Count data are expressed as frequency constituent ratio. ANOVA test was used to compare continuous data between groups, and chi-squared and nonparametric test were applied to test incontinuous and categorical data, respectively. Partial correlation coefficients were used to assess the relationship between serum TSH and sleep and biochemical parameters. Multiple linear regressions analysis was used to assess the associations between serum levels of TSH and AHI, LSaO_2_, ODI_3_, ODI_4_, BMI, age, gender, and systolic and diastolic blood pressure. Value of *P* < 0.05 was considered to be statistically significant. The statistic analyses were performed using the program SPSS (version 17.0).

## 3. Results

### 3.1. Demographic Characteristics

Mean age and BMI of total subjects were 45.59 ± 9.58 years and 28.04 ± 4.02 kg/m^2^, respectively. Prevalence of overweight and obesity was 40.62% and 47.39%, respectively. OSA was diagnosed in 373 patients (72.15%) who were referred to as the OSA group, mild, moderate, and severe OSA constituting 28.82%, 21.66%, and 21.66%, respectively. The other 144 subjects confirmed to have no OSA were classified as the control group.

### 3.2. Thyroid Disorders

Overt hyperthyroidism was diagnosed in 2 subjects in moderate and severe OSA groups, respectively, and subclinical hyperthyroidism in 7 participants in non-OSA (*n* = 1) and in mild (*n* = 4), moderate (*n* = 1), and severe OSA group (*n* = 1), respectively. Overt hypothyroidism was diagnosed in 5 subjects in non-OSA (*n* = 1) and mild (*n* = 1), moderate (*n* = 1), and severe OSA group (*n* = 2), respectively. Prevalence of subclinical hypothyroidism showed an increasing trend across non-OSA groups to various OSA groups and was 6.94% in non-OSA (*n* = 10), 13.4% in mild OSA (*n* = 20), 15.2% in moderate OSA (*n* = 17), and 17% in severe OSA subjects (*n* = 19) and reached a significant difference between non-OSA and overall OSA subjects (6.9% versus 15.0%, *P* = 0.014).

### 3.3. Comparison of Clinical Characteristics, Sleep Parameters, and Thyroid Hormones between Groups

As in Table 1, significant difference exists in gender distribution among groups. Hypertensives with OSA were significantly older, compared to non-OSA ones, and mean ages of OSA subjects were parallel to severity of OSA. BMI of hypertensives with OSA was significantly larger than that of those without OSA, elevating with severity of OSA, and the largest in severe OSA ones. SBP and DBP were elevated with aggregating OSA severity, the highest in severe OSA subjects, with significant differences, compared to non-OSA, mild OSA (both SBP and DBP), and moderate OSA subjects (only DBP). As expected, LnAHI, LnODI_3_, and LnODI_4_ significantly increased and LnLSaO_2_ significantly decreased with severity of OSA worsening. Of note, severe OSA group had the highest serum LnTSH compared to other groups and showed significant difference, compared to non-OSA subjects (0.99 ± 0.81 versus 0.74 ± 0.77 mIU/L, *P* = 0.009), but other parameters of thyroid function showed no significant differences among groups. Severe OSA subjects showed significantly higher LnALT, LnGLT, LnBUN, LnCr, LnHs-CRP, and FBG, than did non-OSA and mild OSA subjects.

### 3.4. Correlation between Serum LnTSH and Hypoxia Parameters in Hypertensive Patients

As given in Table 2, partial correlation analysis was performed via controlling for confounders such as age, gender, and BMI, and significant correlation was observed between serum LnTSH and LnAHI (*r* = 0.104, *P* = 0.021), and LnLSaO_2_ (*r* = −0.100, *P* = 0.025) but not between serum LnTSH and LnODI_3_ (*r* = 0.051, 0.256), ODI_4_ (*r* = 0.066, *P* = 0.142), SBP, DBP, LnFBG, LnHs-CRP, LnALT, LnAST, LnGLT, LnBUN, and LnCr.

#### 3.4.1. Multiple Linear Logistic Regression Analysis between LnTSH and Confounders in Hypertensive Population

As in Table 3, multiple linear logistic regression analysis was performed via LnTSH as dependent variable and gender, age, BMI, SBP and DBP, AHI, LSaO_2_, ODI_3_, and ODI_4_ as independent variables and AHI (*P* = 0.036) and LSaO_2_ (*P* = 0.030) were independently associated with serum TSH across total study population. Furthermore, multiple linear logistic regression analysis showed that age might also exert some effects in increased serum TSH, and thus total subjects were subdivided into three groups as subjects aged <35 years, 35 ≤ age ≤ 60 years, and >60 years, and multiple linear logistic regression analysis was further performed via variables used as given above, and it was found that LSaO_2_ (*B* = −0.008, *P* = 0.014), AHI (*B* = 0.005, *P* = 0.007), ODI_3_ (*B* = 0.003, *P* = 0.044), and ODI_4_ (*B* = 0.003, *P* = 0.025) were independently associated with serum TSH in those aged between 30 and 65 years.

### 3.5. Distribution of Serum TSH in Non-OSA and OSA Subjects

Previous research showed that the incidence of thyroid dysfunction in Chinese with TSH between 1.0 and 1.9 *μ*IU/mL was the lowest and recommended as a safe range [21], and since TSH ≥ 4.5 *μ*IU/mL is the cutoff recommended by guidelines to diagnose hypothyroidism [20], total subjects were divided into four groups as TSH < 1.0 *μ*IU/mL (*n* = 42), 1.0 ≤ THS ≤ 1.9 *μ*IU/mL (*n* = 153), 1.91 ≤ TSH < 4.5 *μ*IU/mL (*n* = 234), and TSH ≥ 4.5 *μ*IU/mL (*n* = 71) and of note, only 26.3% of OSA subjects exhibited serum TSH of 1.0 and 1.9 *μ*IU/mL, significantly lower than did non-OSA subjects (26.3% versus 38.2%, *P* = 0.01), and as given in Table 4 we further compared some demographic characteristics, polysomnographic data, renal and hepatic biomarkers, FBG, and lipid profiles and observed that subjects with TSH ≥ 4.5 *μ*IU/mL were significantly older than were subjects with 1.0 ≤ THS ≤ 1.9 *μ*IU/mL and 1.91 ≤ TSH < 4.5 *μ*IU/mL. BMI of subjects with TSH ≥ 4.5 *μ*IU/mL was significantly higher than that of those with 1.91 ≤ TSH < 4.5 *μ*IU/mL and surprisingly, subjects with TSH < 1.0 *μ*IU/mL showed the highest BMI. DBP was increased with TSH increasing from subjects with 1.0 ≤ THS ≤ 1.9 *μ*IU/mL to TSH ≥ 4.5 *μ*IU/mL and the highest in subjects with TSH ≥ 4.5 *μ*IU/mL, significantly differing from DBP of those with 1.91 ≤ TSH < 4.5 *μ*IU/mL (96.32 ± 14.19 versus 92.31 ± 12.86 mmHg, *P* = 0.04). In lipid profiles, LnLDL-c and LnTG showed an increased tendency across subjects with 1 ≤ TSH ≤ 1.9 *μ*IU/mL to those with TSH ≥ 4.5 *μ*IU/mL, but only LDL-c was significantly different between subjects with 1 ≤ TSH ≤ 1.9 *μ*IU/mL to those with TSH ≥ 4.5 *μ*IU/mL (0.99 ± 0.60 versus 0.87 ± 0.34 mmol/L, *P* = 0.023). Surprisingly, LnTC decreased and LnHDL-c increased with TSH increasing within entire subjects, and LnTC of subjects with TSH ≥ 4.5 *μ*IU/mL was significantly lower than that of those with 1 ≤ TSH < 1.9 *μ*IU/mL and with TSH < 1.0 *μ*IU/mL. LnHDL-c of subjects with TSH ≥ 4.5 *μ*IU/mL was significantly higher than that of those with 1 ≤ TSH ≤ 1.9 *μ*IU/mL and with TSH < 1.0 *μ*IU/mL.

## 4. Discussion

Subclinical hypothyroidism is defined as TSH concentration ≥ 4.5 *μ*IU/mL, while FT_4_ concentration is normal [20]. Increased serum TSH is well established to correlate with hypertension, cardiovascular diseases, and metabolic disorders [3–5]. Our primary findings encompass the following: (1) prevalence of subclinical hypothyroidism is significantly higher in the hypertensives with OSA than in those without OSA; (2) serum LnTSH is significantly increased in hypertensive patients with severe OSA than in those without OSA (Table 1); (3) LSaO_2_, AHI, ODI_3_, and ODI_4_ were observed to be independent risk factors for increased serum TSH in subjects aged between 30 and 65 years even after adjusting for related confounders (Tables 2 and 3); (4) only 26.3% of OSA subjects exhibited serum TSH of 1.0 and 1.9 *μ*IU/mL, significantly lower than did non-OSA subjects; (5) of cardiometabolic risk factors, DBP and LDL-c showed an increased trend with TSH increasing but differed significantly only between euthyroid and subclinical hypothyroid subjects (Table 4).

In the present study, prevalence of subclinical hypothyroidism showed an increasing trend across non-OSA groups to various OSA groups, from 6.94% in non-OSA, 13.4% in mild OSA, and 15.2% in moderate OSA to 17% in severe OSA subjects and with significant difference between hypertensives with non-OSA and OSA (6.94% versus 15.0%, *P* = 0.014) and was somewhat higher than population prevalence approximately 5–10% [22] and than previously reported prevalence in OSA patients [11–16]. TSH, a polypeptide hormone secreted by thyrotrope cells of anterior pituitary, plays a pivotal role in controlling the HPT axis. Tight negative feedback regulation is the characteristic of HPT axis and is the key to using a serum TSH for the diagnosis and management of thyroid diseases, since its changes significantly amplify small changes in T4 [23, 24]. Above findings from the current study possibly suggest that OSA might be a risk factor for increased serum TSH and that a relevant clinical relationship exists between OSA and increased TSH in circulation. This speculation is based on two reasons: (1) increased production of TSH by some stimulating factors in OSA or (2) decreased hepatic and renal clearance and subsequent increase in TSH half-life [25]. However, no establishment of independent relationship between hepatic and renal biomarkers and serum TSH (Tables 2 and 3) suggests that increased TSH is possibly attributable to increased production, indicating OSA might be a risk factor for increased serum TSH.

Underlying mechanisms that may explain the relationship between OSA and increased serum TSH are not fully understood, whereas it is creditable to take into consideration such hall-mark characteristics of OSA as intermittent hypoxia, altered sleep architecture, and systemic inflammation. First of all, hypoxia might play a critical role in increased serum TSH. Previous studies reported serum TSH of subjects exposed to acute hypobaric hypoxia was significantly increased, compared to their sea-level counterparts and ascribed to possible alteration in pituitary negative feedback set point for secretion of TSH [26]. In turn, in a parallel randomized sham placebo controlled trial, one month of continuous positive airway pressure significantly reduced serum TSH in active treatment group compared to pretreatment serum TSH [27]. Furthermore, qualitative and quantitative sleep alterations have been demonstrated to increase TSH, whereas sleep has been demonstrated to have an acute inhibitory effect on overnight TSH secretion [28]. TSH of subjects experiencing sleep withdrawal or partial sleep deprivation late in the night augmented nocturnal TSH secretion and recovered sleep suppressed circadian variation and reversed TSH [22, 29]. Moreover, chronic low grade inflammation such as obesity and OSA is a commonly accepted risk for autoimmune thyroiditis such as Hashimoto's thyroiditis which is the most common cause of elevated TSH and highly prevalent in OSA [1, 13, 22], as supported by the evidence that long-term sleep apnea increases the risk of cell mediated autoimmunity [29–31].

A prospective survey for Chinese population showed that incidence of thyroid dysfunction for serum TSH 1.0–1.9 *μ*IU/mL was the lowest and TSH >1.9 *μ*IU/mL predispose population to hypothyroidism and <1.0 *μ*IU/mL to hyperthyroidism [22]. Therefore, we divided total subjects into four groups as TSH < 1.0 *μ*IU/mL, 1.0 ≤ THS ≤ 1.9 *μ*IU/mL, 1.91 ≤ TSH < 4.0 *μ*IU/mL, and TSH ≥ 4.5 *μ*IU/mL and found that only 26.3% of OSA subjects exhibited serum TSH between 1.0 and 1.9 *μ*IU/mL, significantly lower than did non-OSA subjects, possibly suggesting that OSA is risk factor for increased TSH even within the reference range. Of cardiometabolic risk factors, DBP and LDL-c also showed an increased trend and significantly differed only between groups with 1 ≤ TSH < 1.9 and TSH ≥ 4.5 *μ*IU/mL, and thus it might be reasonable to speculate that increased TSH in OSA may exert some negative effects on lipid profiles.

The current study, however, contains some limitations. Thyroid function tests were performed only once, which may have increased false positivity in current study subjects since the nocturnal peak of serum TSH may be delayed in those with irregular sleep patterns [32]. However, the strength of this study was the large number of investigated subjects, allowing for the analysis of detecting small effects and the quite strict inclusion and exclusion criteria used. Moreover, characteristics of the study design, cross-sectional one, did not allow us to draw a causal relationship between OSA and increased serum TSH. In conclusion, we showed in accordance with previous studies that serum TSH levels are increased in some hypertensive Chinese with severe OSA and more than a half of hypertensives with OSA showed a serum TSH out of recommended safe range, possibly suggesting OSA is a risk factor for increased TSH. Increased serum TSH is associated with some of the cardiometabolic risk factors and the small TSH changes, if chronic, may contribute to the development of disease conditions [7, 13], and thus performing thyroid function testing in hypertensives with OSA is reasonable.

## Figures and Tables

**Table 1:** Comparison of demographic characteristics and polysomnographic and biochemical parameters between non-OSA and mild to severe OSA subjects.

	Non-OSA (*N* = 144)	Mild OSA (*N* = 149)	Moderate OSA (*N* = 112)	Severe OSA (*N* = 112)
Gender (M/F)	87/57	95/54	86/26^bd^	95/17^ce^
Age (years)	42.39 ± 9.15	45.89 ± 9.59^a^	48.22 ± 10.02^bd^	46.66 ± 8.61^c^
BMI (kg/m^2^)	26.62 ± 3.50	28.00 ± 4.20^a^	28.28 ± 3.81^b^	29.72 ± 3.96^cef^
SBP (mmHg)	137.10 ± 18.35	138.81 ± 19.41	140.04 ± 19.54	144.41 ± 18.24^ce^
DBP (mmHg)	93.11 ± 11.90	94.09 ± 13.68	94.93 ± 14.48	98.73 ± 13.68^cef^
LnAHI (events/h)	0.35 ± 0.96	2.14 ± 0.31^a^	3.05 ± 0.19^bd^	3.91 ± 0.33^cef^
LnLSaO_2_ (%)	4.46 ± 0.11	4.39 ± 0.07^a^	4.33 ± 0.08^bd^	4.16 ± 0.24^cef^
LnODI_3_ (events/h)	2.54 ± 0.64	3.33 ± 0.39^a^	3.80 ± 0.36^bd^	4.38 ± 0.26^cef^
LnODI_4_ (events/h)	1.55 ± 0.89	2.73 ± 0.49^a^	3.43 ± 0.34^bd^	4.20 ± 0.31^cef^
LnTSH (*μ*IU/mL)	0.74 ± 0.77	0.87 ± 0.76	0.83 ± 0.75	0.99 ± 0.81^c^
FT_3_ (pg/mL)	3.54 ± 1.56	3.25 ± 0.75	4.99 ± 3.44	4.01 ± 4.20
FT_4_ (ng/dL)	6.60 ± 6.37	6.12 ± 6.94	7.70 ± 8.10	7.28 ± 8.56
TT_3_ (ng/mL)	1.40 ± 0.35	1.41 ± 1.74	1.40 ± 0.40	1.38 ± 0.49
TT_4_ (ng/mL)	39.96 ± 40.08	35.03 ± 31.91	41.31 ± 40.87	40.01 ± 39.07
ATG (IU/mL)	10.02 (6.13)	10.89 (7)	11 (6.56)	11 (5)
TPO (IU/mL)	10 (15)	9.09 (16.05)	10 (15)	9.82 (15)
LnALT (U/L)	3.26 ± 0.56	3.30 ± 0.58	3.23 ± 0.46	3.40 ± 0.52^cf^
LnAST (U/L)	3.01 ± 0.41	3.03 ± 0.40	2.98 ± 0.35	3.11 ± 0.42^f^
LnGLT (U/L)	3.42 ± 0.67	3.57 ± 0.69	3.60 ± 0.71	3.77 ± 0.71^ce^
LnCr (*μ*mol/L)	4.20 ± 0.33	4.20 ± 0.24	4.25 ± 0.23	4.27 ± 0.24^ce^
LnBUN (mmol/L)	1.62 ± 0.27	1.62 ± 0.24	1.69 ± 0.26	1.70 ± 0.38^ce^
LnHs-CRP (mg/L)	0.068 ± 1.09	0.32 ± 0.95^a^	0.15 ± 0.99	0.52 ± 1.01^cf^
FBG (mmol/L)	4.99 ± 0.77	5.13 ± 1.33	5.27 ± 1.49	5.66 ± 1.88^cef^

^a^Control versus mild OSA; ^b^control versus moderate OSA; ^c^control versus severe OSA; ^d^mild versus moderate OSA; ^e^mild versus severe OSA; ^f^moderate versus severe OSA. Significance: *P* < 0.05.

BMI: body mass index; SBP: systolic blood pressure; DBP: diastolic blood pressure; AHI: apnea hypopnea index; LSaO_2_: lowest saturation of oxygen; ODI_3_: oxygen desaturation index of 3%; ODI_4_: oxygen desaturation index of 4%; FBG: fasting blood glucose; ALT: alanine aminotransferase; AST: aspartate transaminase; GLT: glutamic transaminase; Cr: creatinine; BUN: blood urea nitrogen; Hs-CRP: high-sensitivity C-reactive protein; TSH: thyroid stimulating hormone; FT_3_: free triiodothyronine; FT_4_: free thyroxine; ATG: thyroglobulin; TPO: thyroid peroxidase.

**Table 2:** Relationship between serum LnTSH and TSH risk factors and polysomnographic measures controlled for age, gender, and BMI.

Variables	Hs-CRP	LnGLT	SBP	DBP	LnLSaO_2_	LnAHI
*r*	0.018	0.037	−0.002	0.013	−0.100	0.104
*P*	0.686	0.420	0.965	0.768	0.025	0.021

Variables	LnAST	LnALT	LnODI_3_	LnODI_4_	LnCr	LnBUN

*r*	0.039	−0.025	0.051	0.066	0.028	0.031
*P*	0.378	0.572	0.256	0.142	0.538	0.489

BMI: body mass index; Ln: log transformed; TSH: thyroid secreting hormone; AHI: apnea/hypopnea index; LSaO_2_: lowest oxyhemoglobin saturation; ODI_3_: oxygen desaturation index of 3%; ODI_4_: oxygen desaturation index of 4%; FBG: fasting blood glucose; Hs-CRP: high-sensitivity C-reactive protein; Cr: creatinine; BUN: blood urea nitrogen; ALT: alanine aminotransferase; AST: aspartate transaminase; GLT: glutamic transaminase.

**Table 3:** Multiple linear regression analysis between serum TSH and confounders in hypertensives.

	Total subjects			Age < 35 years			35 ≤ age ≤ 60 years			Age > 60 years		
	*B*	Standardized coefficient	*P*	*B*	Standardized coefficient	*P*	*B*	Standardized coefficient	*P*	*B*	Standardized coefficient	*P*
Constant	1.244	—	0.034	−1.010	—	0.343	1.771	—	0.001	7.494	—	0.160
Gender	0.016	0.009	0.839	0.251	0.200	0.180	0.048	0.030	0.549	−0.746	−0.284	0.212
Age	0.010	0.122	0.012									
BMI	−0.008	−0.043	0.349	−0.012	−0.101	0.499	−0.010	−0.053	0.291	−0.006	−0.016	0.943
SBP	0.000	−0.024	0.724	−0.002	−0.088	0.659	0.000	−0.010	0.895	−0.022	−0.280	0.286
DBP	0.001	0.017	0.809	0.009	0.238	0.242	0.000	−0.003	0.972	0.004	0.035	0.906
LSaO_2_	−0.007	−0.103	0.030	−0.015	−0.294	0.067	−0.008	−0.125	0.014	−0.029	−0.233	0.311

Constant	0.560	—	0.203	0.302	—	0.668	1.025	—	0.008	3.357	—	0.344
Gender	0.032	0.019	0.687	0.223	0.177	0.228	0.071	0.044	0.383	−0.598	−0.228	0.344
Age	0.010	0.124	0.010									
BMI	−0.009	−0.045	0.335	−0.011	−0.090	0.542	−0.011	−0.062	0.226	0.014	0.037	0.861
SBP	−0.001	−0.025	0.716	−0.003	−0.131	0.511	0.000	−0.012	0.880	−0.025	−0.326	0.224
DBP	0.001	0.021	0.763	0.011	0.294	0.154	0.000	0.001	0.990	0.020	0.160	0.561
AHI	0.004	0.101	0.036	0.009	0.357	0.070	0.005	0.140	0.007	0.017	0.187	0.377

Constant	0.446	—	0.312	0.466	—	0.506	0.982	—	0.011	3.154	—	0.379
Gender	0.008	0.005	0.915	0.230	0.183	0.212	0.062	0.039	0.447	−0.812	−0.309	0.180
Age	0.011	0.136	0.006									
BMI	−0.005	−0.027	0.571	−0.010	−0.088	0.550	−0.010	−0.053	0.305	0.040	0.105	0.636
SBP	−0.001	−0.027	0.686	−0.003	−0.127	0.523	0.000	−0.012	0.876	−0.019	−0.244	0.368
DBP	0.002	0.031	0.666	0.010	0.267	0.186	0.000	0.000	0.999	0.016	0.127	0.646
ODI_3_	0.001	0.035	0.478	0.006	0.351	0.179	0.003	0.106	0.044	−0.006	−0.158	0.442

Constant	0.501	—	0.256	0.365	—	0.604	1.004	—	0.009	3.343	—	0.359
Gender	0.017	0.010	0.829	0.230	0.183	0.212	0.062	0.039	0.445	−0.812	−0.309	0.189
Age	0.010	0.131	0.007									
BMI	−0.007	−0.035	0.452	−0.009	−0.080	0.587	−0.010	−0.055	0.283	0.029	0.076	0.735
SBP	−0.001	−0.027	0.691	−0.003	−0.130	0.514	0.000	−0.012	0.875	−0.021	−0.274	0.315
DBP	0.002	0.027	0.701	0.010	0.276	0.175	0.000	0.001	0.985	0.018	0.148	0.596
ODI_4_	0.002	0.065	0.180	0.007	0.351	0.132	0.003	0.117	0.025	−0.003	−0.059	0.776

BMI: body mass index; SBP: systolic blood pressure; DBP: diastolic blood pressure; AHI: apnea hypopnea index; LSaO_2_: lowest saturation of oxygen; ODI_3_: oxygen desaturation index of 3%; ODI_4_: oxygen desaturation index of 4%.

**Table 4:** Comparison of demographic and polysomnographic characteristics and biochemical markers in subjects with different serum TSH levels.

	1 ≤ TSH ≤ 1.9 (*N* = 137)	TSH < 1 (*N* = 45)	1.91 ≤ TSH < 4.5 (*N* = 264)	TSH ≥ 4.5 (*N* = 71)
Age (years)	44.59 ± 8.60	47.00 ± 10.57	44.57 ± 9.20	50.49 ± 10.80^cf^
BMI (kg/m^2^)	27.90 ± 3.49	29.33 ± 4.21	27.70 ± 3.58^d^	28.87 ± 5.86^f^
SBP (mmHg)	140.79 ± 20.26	140.10 ± 21.00	139.09 ± 17.82	140.11 ± 19.51
DBP (mmHg)	92.31 ± 12.86	92.62 ± 14.88	95.38 ± 13.41	96.32 ± 14.19^c^
LnAHI (events/h)	2.00 ± 1.55	2.36 ± 1.31	2.28 ± 1.38	2.64 ± 1.25^c^
LnLSaO_2_ (%)	4.38 ± 0.13	4.31 ± 0.22	4.34 ± 0.18	4.31 ± 0.18^c^
LnODI_3_ (events/h)	3.32 ± 0.87	3.63 ± 0.71^a^	3.41 ± 0.81	3.66 ± 0.63^cf^
LnODI_4_ (events/h)	2.71 ± 1.19	3.12 ± 0.98^a^	2.84 ± 1.16	3.20 ± 0.87^cf^
FBG (mmol/L)	5.24 ± 1.52	5.32 ± 1.43	5.29 ± 1.44	5.02 ± 0.92
LnALT (U/L)	3.32 ± 0.56	3.33 ± 0.50	3.31 ± 0.55	3.16 ± 0.48^cf^
LnAST (U/L)	3.04 ± 0.366	2.96 ± 0.31	3.05 ± 0.45	3.00 ± 0.34
LnGLT (U/L)	3.41 ± 0.72	3.54 ± 0.69	3.59 ± 0.70	3.68 ± 0.68^c^
LnCr (*μ*mol/L)	4.25 ± 0.31	4.22 ± 0.26	4.21 ± 0.25	4.21 ± 0.23
LnBUN (mmol/L)	1.68 ± 0.36	1.65 ± 0.30	1.63 ± 0.24	1.69 ± 0.27
LnTC (mmol/L)	1.49 ± 0.26	1.44 ± 0.29	1.42 ± 0.43	1.30 ± 0.57^cf^
LnHDL-c (mmol/L)	0.13 ± 0.32	0.07 ± 0.37	0.21 ± 0.53	0.30 ± 0.50^ce^
LnLDL-c (mmol/L)	0.87 ± 0.34	0.92 ± 0.30	0.92 ± 0.27	0.99 ± 0.60^c^
LnTG (mmol/L)	0.62 ± 0.57	0.73 ± 0.45	0.68 ± 0.58	0.68 ± 0.52
LnHs-CRP (mg/L)	0.28 ± 1.00	0.34 ± 0.96	0.22 ± 1.06	0.27 ± 0.97

^a^1 ≤ TSH ≤ 1.9 group versus TSH < 1 group; ^b^1 ≤ TSH ≤ 1.9 group versus 1.91 ≤ TSH < 4.5 group; ^c^1 ≤ TSH ≤ 1.9 group versus TSH ≥ 4.5 group; ^d^TSH < 1 group versus 1.91 ≤ TSH < 4.5 group; ^e^TSH < 1 group versus TSH ≥ 4.5 group; ^f^1.91 ≤ TSH < 4.5 group versus TSH ≥ 4.5 group. Significance: *P* < 0.05.

BMI: body mass index; SBP: systolic blood pressure; DBP: diastolic blood pressure; Ln: log transformed; AHI: apnea hypopnea index; LSaO_2_: lowest saturation of oxygen; ODI_3_: oxygen desaturation index of 3%; ODI_4_: oxygen desaturation index of 4%; FBG: fasting blood glucose; ALT: alanine aminotransferase; AST: aspartate transaminase; GLT: glutamic transaminase; Cr: creatinine; BUN: blood urea nitrogen; TC: total cholesterol; HDL-C: high density lipoprotein cholesterol; LDL-C: low density lipoprotein cholesterol; TG: triglyceride; Hs-CRP: high-sensitivity C-reactive protein.
